# Navigation as a system approach: A qualitative descriptive study to inform a statewide cancer navigation approach in Australia

**DOI:** 10.1007/s00520-025-09201-6

**Published:** 2025-02-06

**Authors:** Oluwaseyifunmi Andi Agbejule, Ria Joseph, Sue Merchant, Jolyn Johal, Imogen Ramsey, Jacqueline L. Bender, Cally Jennings, Michael Osborn, Fiona Crawford-Williams, Raymond J. Chan

**Affiliations:** 1 Caring Futures Institute, College of Nursing and Health Sciences, Flinders University, Adelaide, SA Australia; 2 Commission on Excellence and Innovation in Health, SA Health, Adelaide, SA Australia; 3 Department of Supportive Care, Princess Margaret Cancer Centre, University Health Network, Toronto, ON Canada; 4 Dalla Lana School of Public Health and Institute of Health Policy, Management, and Evaluation, University of Toronto, Toronto, ON Canada

**Keywords:** Navigation, Cancer, Consumer, Supportive care, Model, Australia, Oncology, Nursing, Cancer survivor

## Abstract

**Purpose:**

This study aimed to identify challenges and facilitators in accessing cancer care in South Australia, from the perspectives of cancer survivors and caregivers, to inform responsive cancer navigation approaches.

**Methods:**

A qualitative descriptive study was conducted using an online qualitative survey (*n* = 75) and video, phone, and in-person semi-structured interviews (*n* = 22) with cancer survivors and caregivers (herein cancer consumers). Data analysis was performed in two phases: content analysis categorised consumer challenges and facilitators, while a subjective-inductive approach guided by the supportive care framework was used to develop a statewide navigation approach.

**Results:**

Key challenges reported by consumers included perceived invalidation of medical concerns, delayed diagnoses, poor communication, inadequate information provision, fragmented care, and limited logistical, cultural, and psychological support. Inductive analysis identified four key themes: 1) cancer consumers have dynamic care needs that can evolve throughout a patient’s cancer experience, 2) cancer consumers require a foundational level of information to support navigation, 3) some cancer consumers express a preference for community-based navigation services to help them manage their care, and 4) individuals with more complex care needs may require more intensive professional navigation services. A conceptual needs-based navigation approach (the Flinders Needs-Based Approach to Cancer Navigation) was developed based on these insights. This approach consists of three levels of navigation interventions: level 1 involves providing information-based navigation to all individuals affected by cancer, level 2 involves community-based navigation support offered to those requiring or wanting additional supported assistance, and level 3 offers professional navigation for individuals with complex needs.

**Conclusion:**

Our study highlights the importance of tailoring cancer navigation services to meet the evolving needs of patients, emphasising the role of both community and professional support, particularly for individuals with complex care requirements. Findings will inform further co-design discussions involving consumers, health professionals, and policymakers to implement cancer navigation services across South Australia.

**Supplementary Information:**

The online version contains supplementary material available at 10.1007/s00520-025-09201-6.

## Introduction

As cancer treatment and diagnostic technologies advance, the landscape of cancer continues to change with people living longer and having ongoing bio-psycho-social needs. Timely and integrated supportive cancer care is pivotal to enhancing cancer survivors’ quality of life and alleviating symptom burdens [1]. Understanding the multifaceted landscape of supportive care needs and experiences, and the complexity of the care ‘journey’, among individuals affected by cancer represents a critical step towards enhancing cancer care. However, conventional cancer care descriptions often depict a linear, health service-focused continuum, emphasising biomedical aspects and the ‘ideal’ services and treatments provided by providers at each phase of care [2, 3]. While informative, this categorisation of the cancer care continuum often overlooks the intricate lived experience of individuals with cancer, including the logistical, financial, psychosocial, and physical complexities associated with diagnosis and accessing care [4–6]. Understanding how individuals affected by cancer experience the cancer care continuum is crucial for identifying gaps in care and developing effective solutions to address these complex needs. Moreover, these complexities can hinder access to care, resulting in inadequate or inequitable allocation of health resources and reduced engagement with essential healthcare services [7].

Cancer navigation refers to individualised, patient-centred, pathways, interventions, or services that aim to overcome patient and system-level barriers to accessing cancer care [7]. This can encompass education and information provision; care coordination, direct care provision, psychosocial support, advocacy, financial, and logistical assistance. The significance and impact of navigation in cancer care have been extensively established. A recent umbrella review [7] highlighted the effectiveness of cancer navigation interventions on improved access to care, reduced waiting times for diagnosis and treatment, increased participation in cancer screening, enhanced care coordination; improved care continuity as evidenced by higher rates of referrals and adherence to follow-up appointments, increased completion of screening and diagnosis, and reduced hospital readmission and emergency visit rates [7].

While individual navigation programs have been shown to be beneficial, the evidence is often organisation-specific, with little information on how navigation can be integrated into a system-wide approach. Further information is needed on how cancer navigation can be applied at a broader, system-level to ensure cohesive and integrated navigation support across an entire health system. Moreover, while various models of navigation have existed in North America for over a decade, other countries may currently implement components of navigation without sharing the same definitions and approaches [7]. This research study was conducted to inform a state-wide cancer navigation approach with the aim of improving equity of cancer care delivery in South Australia. By exploring the experiences and challenges in cancer care in South Australia, through the eyes of individuals affected by cancer (i.e., cancer survivors and their caregivers—herein cancer consumers), we endeavoured to identify valuable insights to inform cancer navigation approaches. The specific objectives of this research were to 1) identify the gaps and challenges encountered by individuals navigating cancer care in South Australia, 2) explore the factors that may have enhanced their care experiences, and 3) use these insights to inform a system-level cancer navigation approach.

## Methods

This study was reported according to the Standards for Reporting Qualitative Research (SRQR). We conducted a qualitative descriptive study [8] that comprised an online qualitative survey [9] followed by virtual and in-person semi-structured interviews with individual consumers. Survey responses were collected concurrently with the interviews. This study employed a two-phase data analysis approach. In phase one, content analysis was used to categorise experiences of consumer challenges and navigation facilitators. In phase two, a sub-group of the research team analysed the raw qualitative data using a subjectivist-inductive approach [10], guided by the theoretical foundations of the supportive care framework [11]. This analysis approach led to the development of a needs-based approach to navigation in South Australia.

### Participants

Individuals were eligible for survey participation if they were at least 18 years old, competent in written and verbal English, and either had a cancer diagnosis or had cared for someone diagnosed with cancer in South Australia.

### Recruitment

Using a combination of purposive and snowball sampling methods, we advertised the study to potential participants via known networks, cancer advocacy organisations, non-government organisations, public hospitals, and cancer support groups across metropolitan and regional South Australia. To reach diverse communities, we engaged with multicultural organisations such as the Multicultural Communities Council of South Australia, the Australian Refugee Association, the Nigerian Association of South Australia, and Indonesian community. Individuals from these consumer organisations and groups subsequently shared study information, and/or a link to the online survey across their memberships and networks. We also translated study information flyers into Vietnamese, Arabic, Mandarin, and French and offered potential participants various options for providing feedback (e.g., individual meetings, group settings, and survey). For non-English speakers, we planned to translate the survey and provide interpreters for interviews, should there be expressions of interest. Sample size was estimated to be sufficient based on the principle of meaning saturation [12], whereby no new information about the meaning of categories/themes and their relationships emerged.

### Materials

We developed an online survey in Qualtrics™ XM to collect information from cancer survivors about their perspectives and experiences of cancer care they received or were currently receiving in South Australia. The survey included multiple choice and open-ended questions, which were categorised according to different timepoints within the cancer care continuum (e.g., identifying consumer experiences before a cancer diagnosis, immediately after diagnosis, during treatment, after treatment, and during survivorship/follow-up). The survey also collected information on the following demographic characteristics: age, ethnicity, sex, primary cancer site of first diagnosis, time since first diagnosis (years), and consumer participant type (e.g., patient or caregiver). For participant interviews, we developed an interview guide (Supplementary 1). Semi-structured interviews were offered to allow each participant to talk further about their experiences across the continuum of care, and further contextualise their survey responses. The interview guide was designed to be used flexibly and tailored to each participant’s responses. Throughout the survey and interview, caregivers were instructed to provide responses relating to any gaps, challenges and facilitators in accessing cancer care experienced by both the patient they cared for and themselves.

### Procedures

#### Phase 1—surveys and interviews

The survey link was disseminated via various individuals and organisations as described above. At the completion of the online survey, participants interested in providing additional information through an interview were invited to provide their contact details, which were stored separately from the anonymous survey responses. These individuals were contacted by the research team to arrange an interview via telephone, videoconference, or in-person depending on interviewee preference. Interviews were audio-recorded and transcribed by the research team, with the written transcripts de-identified.

#### Phase 2—development of cancer navigation approach: sub-group and steering committee

A sub-group of the research team (OAA, RJC, FCW, and RJ) developed the navigation approach (analytic procedures described below). This group comprised a professor in cancer nursing and policy, a public health researcher with expertise in psychology, and two clinician researchers with expertise in self-management, and systems thinking, respectively. All group members had additional expertise in survivorship care, supportive care, cancer navigation, and clinical research. An external steering committee was also established to provide comments and feedback on the developed navigation approach. This committee comprised cancer consumers; members of cancer advocacy groups and peak cancer bodies (e.g., Cancer Voices South Australia), multi-disciplinary health professionals, and representatives from the South Australian Government.

### Data analysis

#### Phase 1—surveys and interviews

Basic descriptive analysis (i.e., mean, median, frequency, and range) was conducted to summarise the demographic profile of both the online qualitative survey and interview participant sample. First, free-text survey responses were analysed and checked by two authors (OAA and SM) using content analysis methods independently [13]. Examples of the content analysis process are presented in Supplementary 2. Briefly, the free-text survey responses were read several times and ‘meaning units’ were identified. Meaning units were then assigned codes, which were compared to identify similarities and differences. Through these comparisons, codes were combined to form categories and subcategories related to the research aims: (1) gaps and challenges in accessing cancer care and (2) facilitators to accessing cancer care. Categories were grouped according to their timepoint in the care continuum and iteratively revised as data collection and analysis progressed. Second, interview data were coded by two authors (OAA and SM) independently according to the categories and themes developed through analysis of the survey data, with any new categories being recorded [14].

Facilitators to accessing cancer care were deductively categorised (across the survey and interview data set) [14] into the nine domains of cancer navigation (i.e., care coordination, education/information provision, empowerment, comfort/ emotional support, direct care provision, advocacy, language assistance, logistics assistance, and financial assistance) outlined in a recent umbrella review [7].

#### Phase 2—conceptualisation of a navigation needs-based approach

A subjectivist, framework-informed inductive analysis method [15] was used to transform the qualitative data into an approach to cancer navigation in South Australia. To ensure sustainable and equitable care with limited resources, it is essential to prioritise navigation support by individual needs [16, 17]. The research team adopted a needs-based approach to cancer navigation, using the Supportive Care Framework [11] as a foundation for data analysis. This framework emphasises that supportive care should align with an individual’s specific needs, goals, and context, rather than the preferences of healthcare providers, making it a suitable framework for subjectivist-inductive data analysis.

Applying this needs-based lens to data analysis, we focused on identifying themes related to participant needs. Specifically, the original uncoded survey and interview data were examined to determine what types of navigation interventions were desired, by whom, and for what reasons. This process involved thematic analysis exploring the nuances of participants’ experiences, such as the reasons behind their preferences for certain types of navigation support and teasing out reasons for factors that facilitated or hindered their cancer care. Based on these insights, an initial needs-based navigation approach was developed and subsequently presented to the steering committee for further review and feedback. This feedback led to further refinements in content, wording, and presentation as additional qualitative data were collected.

## Results

### Phase 1—online survey and semi-structured interviews

#### Summary of participant characteristics

Of the 110 eligible individuals who participated in the online survey, seventy-five respondents (*n* = 52/75 (69.3%) cancer survivors and *n* = 23/75 (30.7%) family members/caregivers) completed at least one question beyond demographics, and thus were included in the survey analysis. Of these 75 respondents, 72 completed the entire survey. Most respondents were over 65 years (48.0%, *n* = 36/75), identified as White or Caucasian (90.7%, *n* = 68/75); and identified as female (54.5%, *n* = 42/75). Of the 52 cancer survivors, most had a primary diagnosis of genitourinary (30.8%, *n* = 16/52) or haematological cancers (26.9%, *n* = 14/52). Full demographics are presented in Table 1. A total of 22 individuals (18 cancer survivors, two caregivers, and two individuals who were both cancer survivors and caregivers) participated in the semi-structured interviews. Most interview participants were White or Caucasian (90.9%, *n* = 20/22), male and over 65 years (72.7%, *n* = 16/22), and diagnosed with a genitourinary (36.3%, *n* = 8/22) or haematological cancer (31.8%, *n* = 7/22). Full demographics are presented in Table 1.

**Table 1:** Survey and interview demographic data

S**urvey general demographics (*N*** **= 75)**		**Interview general demographics (*N*** **= 22)**
	***n* (%)**	***n* (%)**
**Age category (years)**		
18–25	2 (2.7)	
26–35	1 (1.3)	
36–45	1 (9.3)	1 (4.5)
46–55	11 (14.7)	1 (4.5)
56–65	18 (24)	4 (18.2)
Over 65 years	36 (48.0)	16 (72.2)
**Sex**		
Female	42 (56.0)	6 (27.3)
Male	33 (44.0)	16 (72.7)
**Ethnicity**		
White or Caucasian	68 (90.7)	20 (90.9)
First Nations person (Aboriginal or Torres Strait Islander)	4 (6.7)	
Asian	1 (1.3)	1 (4.5)
Biracial or multiracial	1 (1.3)	1 (4.5)
**Consumer type**		
Cancer survivor/patient	52 (69.3)	19 (86.4)
Family member/caregiver	23 (30.7)	3 (13.6)
**Cancer survivor-specific demographics (N = 52)**		**Cancer survivor (*N* = 19)**
Primary cancer site		
Genitourinary	16 (30.8)	6 (31.5)
Haematological	14 (26.9)	8 (42.1)
Brain	5 (9.6)	1 (5.3)
Breast	4 (7.7)	1 (5.3)
Bone	3 (5.8)	1 (5.3)
Colorectal	2 (3.8)	
Gynaecological	2 (3.8)	
Head and Neck	2 (3.8)	
Melanoma	2 (3.8)	2 (10.5)
Unknown primary	1 (1.9)	
Thyroid	1 (1.9)	
**Time since initial diagnosis of primary cancer**		
Less than 1 year	4 (7.7)	
1–2 years	9 (17.3)	
2–5 years	18 (34.6)	
5–10 years	13 (25.0)	
More than 10 years	8 (15.3)	
**Caregiver/family member-specific demographics (N = 23)**		**Caregiver/family (*N* = 3)**
Brain	4 (17.4)	
Colorectal	4 (17.4)	
Breast	3 (13.0)	
Genitourinary	3 (13.0)	1 (33.3)
Haematological	3 (13.0)	
Bone	2 (8.6)	1 (33.3)
Lung	2 (8.6)	
Pectoral muscle	1 (4.3)	1 (33.3)

#### Consumer experience of cancer care in South Australia

The overarching categories derived from participants’ survey responses and interview transcripts (gaps and facilitators to cancer care access) are presented in Table 2. This table combines insights from both caregivers and patients, as experiences were similar for all participant groups.

**Table 2:** Gaps, challenges, and facilitators to accessing cancer care in South Australia

Pre-diagnosis	Diagnosis & early detection	After diagnosis (before treatment)	Treatment	Long-term follow-up
Gaps and challenges in accessing cancer care				
**Communication and information gaps** • Challenges in communication between patient and medical team • Challenges in communication within health care team (between disciplines) • Consumers had low awareness regarding cancer, screening, managing risk factors, and symptoms Perceived invalidation of medical concerns and delayed diagnosis • GPs dismissive of patient concerns • Had to self-seek diagnostic imaging • Perceived delayed diagnosis • Misattribution of symptoms by healthcare providers • Assumptions by medical specialists **Logistical barriers** • Long GP wait times (regional areas) • Poor access to specialist services in regional areas **Poor care coordination** • Delays in care, long GP wait times • Limited continuity of care • GPs possessed limited awareness of treatment options and referral pathways	**Treatment delays and challenges** • Delays in treatment initiation • Delays in medical investigation • Administrative delays in treatment • Long wait times in private clinics • Health care team not proactive **Lack of culturally tailored support** • No information or support provided to familiar support networks **Practical and logistical barriers** • Long GP-wait times • Poor access to specialist services in regional areas • Limited access to personal scans **Communication and information gaps** • Challenges in communication between patients and their medical teams • Challenges in communication within health care team (between disciplines) • Health care teams unaware of different support services	**Communication and information gaps** • Conflicting treatment information • Lack of communication from specialists • Limited information on sexual health and dietary changes after diagnosis • Overwhelming information – too much jargon • Limited awareness of treatment options • Had to self-initiate research on available support services • Absence of ongoing support beyond surgical procedures **Practical and logistical barriers** • Difficulty accessing rehabilitation • Delay in accessing medical care • Challenges in securing hospital beds • Poor appointment scheduling **Financial difficulties** • Ineligibility for support schemes, difficult accessing National Disability Insurance Scheme **Lack of culturally tailored support** Lack of support for First Nations individuals • No support provided to familial network • Lack of bilingual clinicians **Limited emotional and psychological support** • No emotional support *(“just treatment plans”)*	**Communication and information gaps** • Lack of information about treatment side effects • Challenges in understanding and navigating treatment options • Breakdown in communication between health care providers **Limited emotional and psychological support** • Delayed psychiatric support, no culturally tailored support • Lack of empathy and open communication • Psychosocial care not emphasised or included in treatment pathway **Practical and logistical Barriers** • Travel challenges and logistical issues for treatment • Lack of medical and nursing support **Poor information provision and lack of knowledge** • Difficulty dealing with administrative tasks • Lack of awareness about treatment implications • Limited information on clinical trials. Left to the patient to identify • Not understanding treatment options **Fragmented and non-coordinated treatment** • Delay in test results and clinical trial issues • Breakdown in communication between healthcare providers (hospital and home care) • Challenges in accessing complementary treatments • Problem dealing with all the administrative tasks (patient perspective) **Unaddressed carer needs** • Limited psychosocial care offered to family and caregivers • Lack of support for patients who also have caring responsibilities	**Communication and information gaps** • Limited information and guidance on available support services for symptom management, life after cancer, return to work, accessing financial support. Hard to know where to find accurate information • Lack of information about treatment side effects • Breakdown in communication between providers **Challenges in managing side effects** • Difficulty managing side effects • Unawareness of medical terminology and options • Challenges in understanding and navigating treatment options **Fragmented and non-coordinated care** • Difficult transition from private to public care • Lack of follow-up and monitoring post-treatment • Difficulty accessing palliative care • Challenges with scheduling remote consultations and appointments **Limited emotional and psychological support** • Emotional and depression challenges post-treatment • Difficult to identify support service **Lack of logistical support** • Limited advocacy and financial assistance **Unaddressed carers needs** • Lack of services/ support for caregivers
Facilitators to accessing cancer care				
**Education and information provision** • Education to debunk myths and dispel fear around cancer • Links to prevention programs • Up-to-date information on symptoms, treatment side-effects, diagnosis, treatment options • Better education of GPs on cancer pathway **Care coordination** • Person navigator (online or face-to-face). A familiar support person • Direct access to diagnostic test results • Education and teaching tools for GPs **Comfort/ emotional support** • In-person counselling for patients and family • A person support to guide the patient through the severity of the diagnosis • More culturally tailored support Empowerment • Better validation of medical concerns	**Education and information provision** • Healthcare practitioner’s competency resources • Better communication around diagnostic wait times • Patient symptom education and empowerment • Making patient information more accessible across different centres and clinics **Care coordination** • Digital application to help track appointments • Tools to assist in treatment decision making • Access to specialists and diagnostics • Continuity of care • Clinical education/ training • Improved clinician knowledge of- and use of referral pathways **Peer support** • Early access to peer support groups • Improve practitioner awareness of different support groups and their services • Psychological support • Accessible support person	**Education and information provision** • Information on available support groups services • Desire for holistic care and support services • Improved communication and coordination • Accessibility and coordination of services • Digital solutions for information sharing and connecting systems **Logistics assistance** • Accommodation, transport/travel assistance **Comfort/Emotional Support** • Counselling for patients and family • Support for how to provide information to employers • Support for patients and families who must leave home behind to travel to treatment appointments **Care coordination** Digital solutions for information sharing and connecting systems • Digital platform for reporting and monitoring symptoms • Information on expected symptoms – what they are and how to manage them • A support person that can assistance with treatment appointments and coordinating health teams • Point of contact to provide guidance on where to find information useful for navigating care • Coordinated accessible team support that could assist with symptom management	**Emotional support** • Psychological support and counselling for dealing with cancer impact **Education and Information Provision** • Improved education and guidance on side effects, treatment options, management of cancer • Informational support tailored to caregivers • Access to supportive resources and information on supportive services (e.g., travel, parking reimbursements, mental health, employment, rights, peer-groups) • Patient support and empathy • Better communication between clinical teams **Direct Care Provision and Advocacy** • Support with symptoms and side effects that cannot be self-managed • Practical support and assistance on behalf of the patient, peer support **Logistical assistance** • Improving treatment access for regional and rural patients/families • Extending support to family/ caregivers that need to travel with patients (e.g., parking, public transport) **Care coordination** • Improving clinician access to diagnostic results, improving communication across different services and systems • Navigator person support having access to the information **Language assistance** • Help with translating documents, unhealthy reliance of family members to act as interpreters – no support provided to family members **Financial assistance** • Assistance with accessing Centrelink and NDIS	**Education and information provision** • More information on how to navigate life after cancer • Information on available support services • Support and guidance for transitions between public and private health systems **Care coordination/ empowerment** • Better medical history and diagnostic information accessibility • Better awareness of specialised support services • Provision of quality resources on post-treatment care • Ensuring survivorship care plans are easily accessible by patients and their support network • Involvement of peer support group **Emotional support/ advocacy** • Access to counselling support for patients and their family/ support network • Support with return to work • Emotional support and trauma management • Support for fear of cancer recurrence • Coordinator/ person navigator for recovery assistance **Financial assistance** • Support with navigating Centrelink and accessing National Disability Insurance Scheme • Support with maintenance scans and consultation fees – especially important due to patient inability to work

**Fig. 1:**
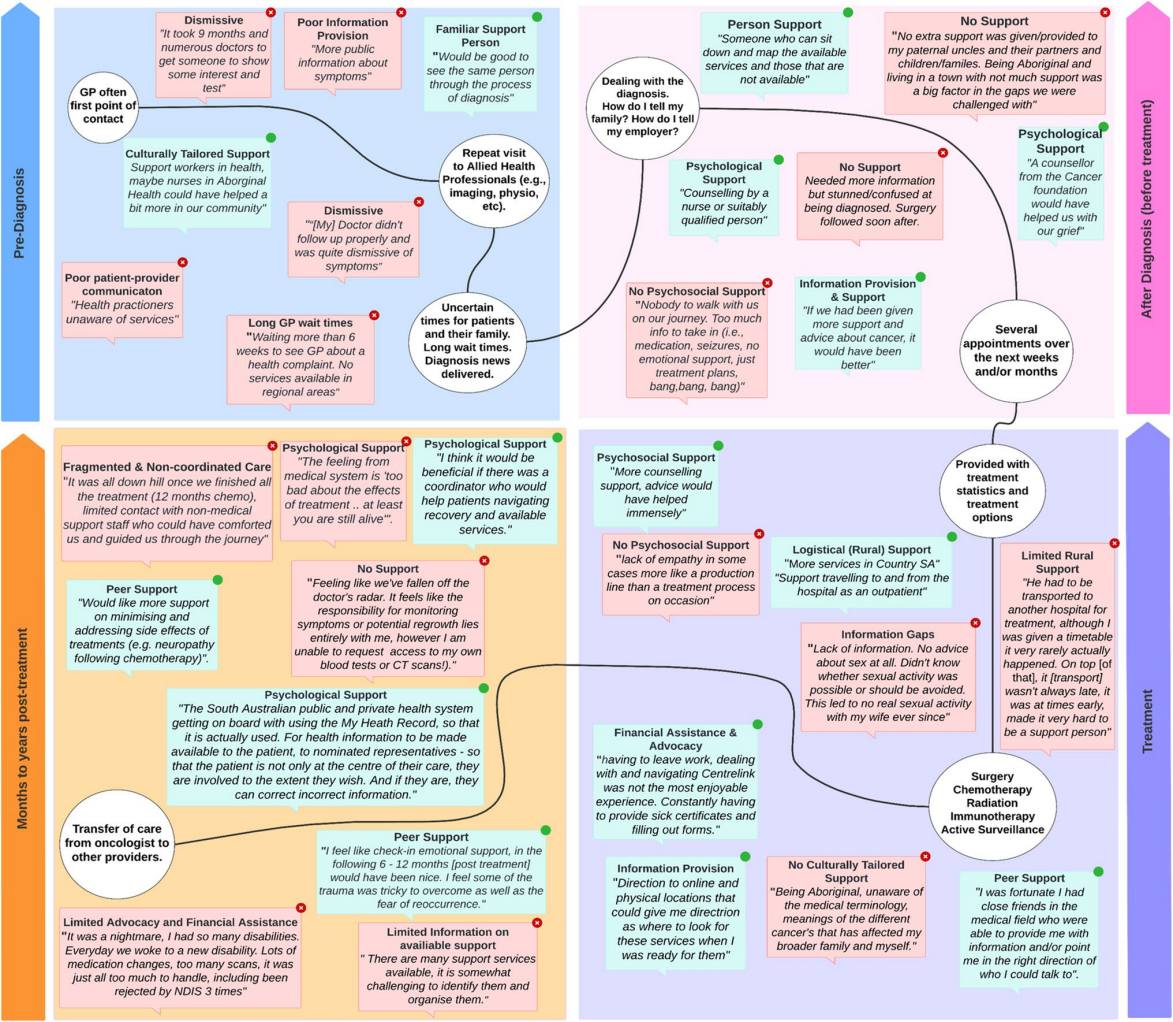
Visual representation of the cancer care continuum from South Australians affected by cancer

Aligning to the major categories and subcategories (presented in Table 2), direct participant quotes were used to present a visual representation of reported gaps and challenges experienced during care (quotes in red) and the potential facilitators/solutions for improved care (quotes in green) at different timepoints (see Fig. 1). Moreover, pre-diagnosis is shown in a blue box, post-diagnosis (before treatment) in a pink box, during treatment in a purple box, and the months to years after treatment in an orange box. The quotations within each box can be read in any order. Titles of quotations are repeated in cases where a particular category was prominent among participants, emphasising the significance of the category in the data. Direct quotes were used to ensure that participants’ first-hand experiences and perspectives were accurately represented.

### Phase 2—informing an approach to cancer navigation in South Australia

**Fig. 2:**
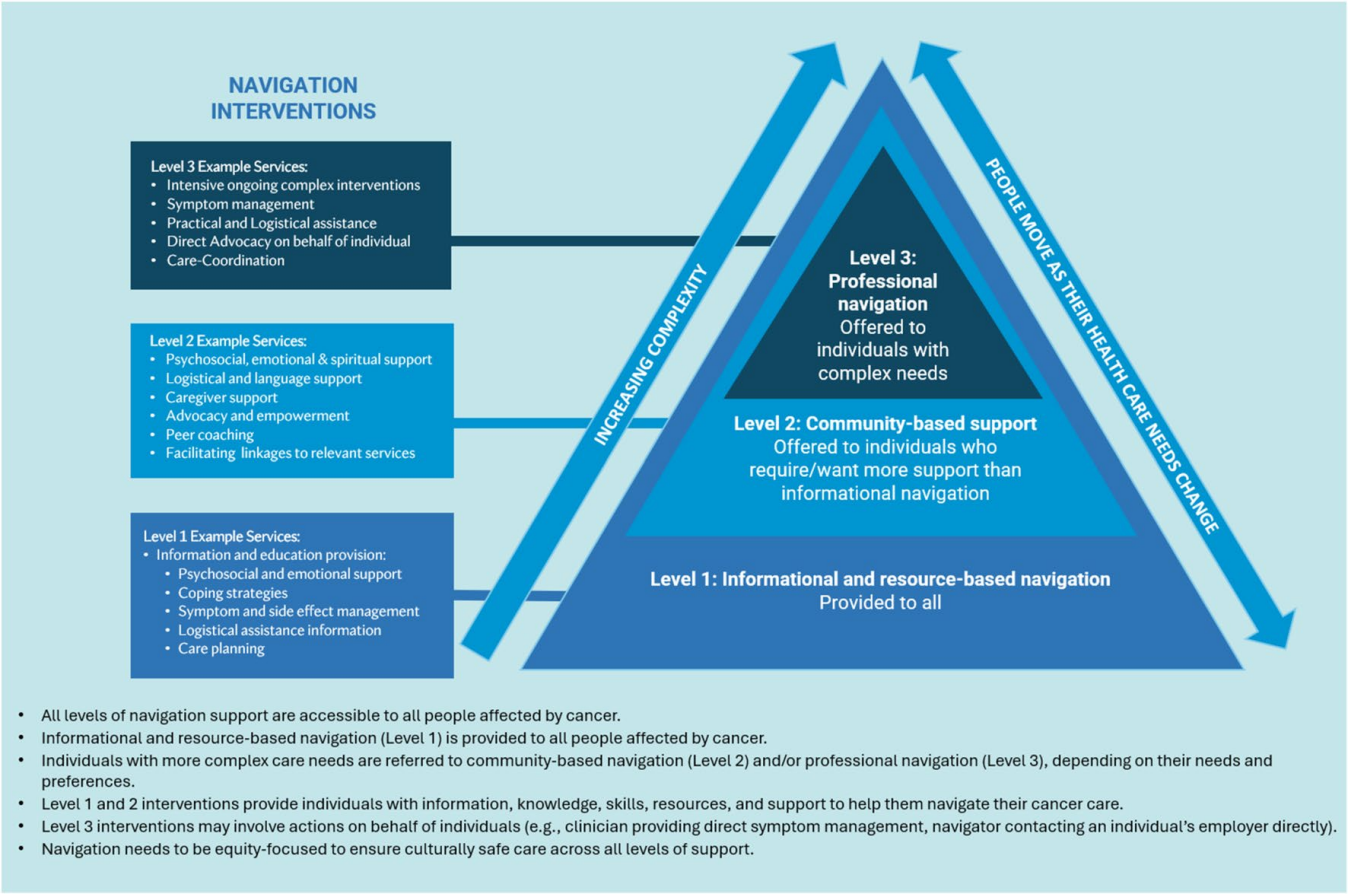
The Flinders Needs-based Approach to Cancer Navigation

Analysis of participant accounts through a needs-based lens resulted in four key themes: 1) dynamic care needs, 2) universal baseline navigation support, 3) additional peer-based support preferences, and 4) priority problems and elevated needs (see Table 3). Supplementary 3 provides a non-exhaustive list of examples of participant quotes that illustrate each theme. Derived from these themes, the Flinders Needs-based Approach to Cancer Navigation, which acknowledges the complexity of an individual’s unique cancer experience is presented below (Fig. 2). The navigation interventions shown on the left-hand side of Fig. 2 are based on those identified as facilitators to accessing care, as outlined in Table 1. Additionally, the analysis of participant accounts and themes (as shown below) guided the organization of these interventions into different levels: level 1 comprising informational and resource-based navigation, level 2 focuses on community-based support, and level 3 involves professional navigation.

**Table 3:** Themes from need-based analysis

**1. Dynamic care needs:** The level of an individual’s care needs (and thus their need for cancer navigation support) is dynamic and can evolve during a patient’s cancer experience
**2. Universal baseline navigation support:** All individuals affected by cancer share a baseline level of informational and resource needs and barriers to care, necessitating a minimum level of informational navigation support *(information to support navigation)*
**3. Preferences for community-based navigation: **Some individuals seek and/or require additional support beyond information and express a desire for peer interaction and community-based navigation support
**4. Intensive support for people with complex needs: **Certain populations have disparate cancer outcomes, diverse experiences of cancer care, and complex care needs, signalling a heightened requirement for more intensive navigation support *(professional-based navigation)*

#### Dynamic care needs

Participants reported experiencing gaps and challenges in care at different points. For example, when asked about their support needs at the pre-diagnosis and diagnosis phases of care, some participants expressed that the informational resources they received were sufficient and they did not utilise an available navigator support person, despite being aware of the option. However, these same participants expressed a need for a navigator during the treatment phase of care. This variability in cancer navigation needs *within an individual’s care trajectory* may reflect changes in their challenges or barriers to accessing cancer care services, as well as shifts in their health and subsequent care requirements. This conceptualisation aligns with the supportive care framework which suggests that specific care needs vary from person to person and within the same person over time as the course of a disease and treatment unfolds [11]. Importantly, variation in navigation needs do not always follow a straightforward progression (e.g., people may not need more navigation support as they move further along the care trajectory); as in some cases, participants needed a more intensive form of navigation support for more complex issues during the diagnosis phase of care, and information navigation support during treatment.

Navigation needs also vary *among different individuals*. For instance, while some participants reported they did not require additional navigator support in the pre-diagnosis phase, others found the pre-diagnosis phase especially challenging and considered it a critical time where a navigator was needed. The model’s double-headed arrow (Fig. 2) illustrates the concept of dynamic navigation needs, indicating individuals may shift across levels (1, 2, and 3) as health care needs change.

#### Universal baseline navigation support

The importance of information provision was a dominant theme among participants. Participants reported various informational needs, such as needing to better understand their treatment options, acquiring strategies to manage side effects and coordinate care, and becoming aware of relevant services and available supports. Despite differences in individual needs and preferences, all participants expressed a requirement for information to support navigation and optimise their care experiences (*e.g., “There needs to be more information given to us from the start, so we know what’s happening rather than me having to research everything by myself.”—*Interviewee 11)*.* In fact, our analysis highlighted that even when participants reported they experienced no challenges in navigating their care, it was often because they perceived they had received sufficient and appropriate informational support (e.g., “*it was seamless for us because we were aware of services and all the staff gave us plenty of information”—*survey respondent)*.* These insights suggest that individuals affected by cancer share a baseline level of informational navigation needs. As such, at a minimum, information to support navigation should be provided to all individuals affected by cancer (Level 1 in Fig. 2).

#### Preferences for community-based navigation support

In the Australian context, community-based navigation support refers services external to the acute or primary care health system, and includes community-funded services, clinicians, and/or non-clinical workers offering personalised guidance, advocacy, and information to people affected by cancer [18]. This navigation support often includes a peer-support component. Several participants emphasised the importance and value of community-based navigation support.

*“I’’ve got a lot of written information, pamphlets, brochures, all sorts of stuff and that’s the issue I had. I got all the written stuff, but I didn’t know who to contact because I had questions. You know it took a bit of running around and you know, I spoke to leukaemia foundation. I spoke to Cancer Council. But I’ve noticed they’re double generic… but I need specifics and eventually it was Myeloma Australia that pointed me in the right direction.”*—Interviewee 6

Many participants reported that community-based navigation (level 2 in Fig. 2) often provided by consumer advocacy groups, or non-government organisations, played a crucial role in offering logistical, emotional, and spiritual support (e.g., “*Finding PCFA* [Prostate Cancer Foundation Australia] *and being in the position with a bit of time on my side allowed me to be to one step ahead of the game so to speak”—*survey respondent). Community-based services aided participants in overcoming logistical (e.g., travel and accommodation) and financial barriers and facilitated connections to other essential care services and professionals.

However, participants acknowledged that not all individuals affected by cancer desired or felt the need for additional community-based navigation (e.g., “*I don’t find the [peer support] help or groups very supportive. They’re just a group that gather because they’re a group.”—*survey respondent. Several participants reported not using community-based supports as they found the informational support they had received, (e.g., *“I was given the contact details* [of myeloma specialist nurse] *but I never needed to call them.”—*Interviewee 11) or the support provided by their own personal support network was sufficient.

*“We have a very supportive community here. We’re a very close-knit community. And you know, I just had people that supported us. They bought us meals. They came and sat with [redacted] while I could go out for a little bit and things like that. I’m probably not the best example for needing, you know, using other support services and requiring them.”—*Interviewee 1

Additionally, many participants reported they did not feel the need to engage in community-based navigation at some timepoints, however they appreciated knowing the option was available (e.g., *“I was provided with a support person* [though the community-based group Myeloma Australia] *throughout my care but didn’t use her services. I didn’t feel I needed her. But it was good to know that support was there, if I wanted”—*survey respondent). Nevertheless, our findings indicated that some individuals may need or express a desire for peer interaction and more personalised, in-person or community-based navigation assistance.

#### Intensive support for people with complex needs

Data analysis indicated that people affected by cancer with complex care needs may require intensive cancer navigation assistance beyond information and community-based navigation. In these cases, professional navigators may need to provide more tailored support directly to (or on the behalf of) patients and their support networks. *Professional navigation* encompasses various roles, which can be categorised into clinical and non-clinical. Clinical roles, whether cancer-specific or not, include positions such as advanced practice nurses and social workers, and are often clinic or hospital-based, providing support for symptom management, direct care provision, and care coordination. Non-clinical professional roles are often paid community-based workers who offer logistical and financial support on behalf of the patient. A key difference is that professional navigation (level 3 in Fig. 2) involves taking direct action for the individual, while community-based or information navigation (levels 1 and 2) empowers individuals with knowledge, skills, and confidence to manage their care.

## Discussion

This qualitative study explored and mapped the care experiences of people affected by cancer in South Australia, from pre-diagnosis to follow-up care. We identified participant’s perceived barriers and facilitators to accessing timely care across the cancer continuum and identified four key themes: (1) dynamic care needs, (2) universal baseline navigation support, (3) preferences for community-based navigation, and (4) intensive support for people with complex needs. From these themes, we developed a tiered needs-based approach to navigation service provision, to inform a statewide navigation framework and action plan.

Consumer mapping is recognised as a critical step in designing effective models of care [19, 20]. This was the first study to map consumer interactions with cancer care in the South Australian context. Mapping participants’ experiences within and across cancer care highlighted the potential role for navigation services to enhance the provision of timely, equitable, coordinated, and comprehensive cancer care in South Australia. Cancer survivors and carers reported gaps such as insufficient information, fragmented and uncoordinated care, practical and logistical barriers, lack of emotional and psychological support, and limited culturally tailored services. These deficiencies are consistent with findings from other global contexts [21–23]. Additionally, participants identified information support, emotional support, advocacy, care coordination, and logistical, financial, and language assistance as important enablers of comprehensive and patient-centred cancer care. These findings support the need for further work to establish navigation services in South Australia and will inform responsive strategies for service improvement [19, 20].

Our study highlighted the importance of a needs-based, system-level navigation approach rather than isolated navigation strategies. Consumers emphasised that current support in South Australia can be fragmented and uncoordinated. A system approach to navigation acknowledges that effective cancer care requires not only individual navigation interventions but also cohesive interconnected services that collaborate to achieve optimal outcomes [24]. To facilitate this cohesive navigation system, a consumer assessment of individual navigation needs [25, 26] must be embedded into routine cancer care so that individuals can seamlessly enter the cancer navigation system and be triaged appropriately across all three levels of navigation. As a key theme from our findings is that the navigation needs are dynamic, an appropriate needs-assessment should inform a seamless escalation and de-escalation model that responds to need in *real time* with appropriate and *timely* navigation support. While there are many current and validated supportive care unmet needs tools for cancer patients [27, 28], there is little evidence on their appropriateness to enable real-time responsiveness to support the unmet navigation needs of people affected by cancer. Future research should explore the development, utility, efficacy, and implementation of such tools. Moreover, an escalation and de-escalation system does not yet exist within Australia and should be co-designed with healthcare and consumer stakeholders, with consensus around who is responsible and available for follow-up actions. Such a system could be supported by digital innovations including artificial intelligence (AI) and algorithm-backed pathways [29].

## Strengths and limitations

This study was conducted within the context of South Australia which may limit the generalisability of results to other regions or populations. Additionally, the study sample primarily comprised Caucasian, male, middle-aged individuals, further restricting the applicability of findings to more diverse populations. Although the study’s predominantly White/Caucasian sample aligns with the broader South Australian population (according to 2021 ancestry census data [30]), future exploration in this area should include perspectives of Indigenous Australians and individuals from culturally and linguistically diverse backgrounds. Another potential limitation is that patients with haematological malignancies were overrepresented in the study population when compared to the actual cancer population in South Australia. However, while this group often has complex needs, most haematological cancer patients in this study reported very few gaps and challenges during and after their care. Despite these limitations, this study provides a comprehensive and patient-centric approach to understanding the entire spectrum of cancer survivors’ experiences with cancer care, allowing us to conceptualise personalised, patient-centred care based on needs and values.

## Conclusion

Conceptualisations of the cancer care continuum can sometimes be dominated by the clinical lens and perspectives of healthcare professionals and/or service providers, often overshadowing insights offered by patients, survivors, their families, and caregivers. In recognising this, our study shifts the focus to the experiences and voices of those directly impacted by cancer. Moreover, the identified challenges, navigation faciliators, and navigation needs-based approach presented in this study will be used as the foundation for further structured co-design group discussions with consumers, health professionals, service providers, and policymakers regarding the implementation and operationalisation of navigation services in South Australia.

## Supplementary Information

Below is the link to the electronic supplementary material.

ESM 1 (DOCX 34.6 KB)
