# Supplementary material for: Navigation as a system approach: A qualitative descriptive study to inform a statewide cancer navigation approach in Australia
Source: Support Care Cancer. 2025 Feb 6;33(3):155. doi: 10.1007/s00520-025-09201-6 (PMC11802597; doi:10.1007/s00520-025-09201-6)
Supplement: Supplementary file 1 — (DOCX 34.6 KB) [file 520_2025_9201_MOESM1_ESM.docx]

**Supplementary 1:** Interview guide

- **For patients**: Do you feel like you experienced any gaps and/or challenges in your care?
- **For family and caregivers:** Did the person you cared for or are currently caring for face any difficulties or gaps in their care?
- Can you please describe these gaps and/or challenges?
- What kind of information or support might have helped you during your care?
- How was the transition to ‘normal’ life after leaving the hospital?
  - What support services did you use? What helped you?, At what point would you have found these services most valuable?, How are you coping at the moment?, If there was one key aspect of support, or perhaps a significant gap, what would it be?, If you could fix only one thing, what would it be?, Did you receive any support when you developed symptom X?, Were you given information about X, etc.).

**Supplementary 2:** An example excerpt of the content analysis of qualitative responses.

| **Time since diagnosis (yrs.)** | **Participant Type** | **Raw Data** | **Meaning Unit** | **Condensed meaning unit** | **Code** | **Category** | **Sub-headings/  Sub-categories** |
| --- | --- | --- | --- | --- | --- | --- | --- |
| Before Diagnosis: Do you feel like you experienced any gaps and/ or challenges in your care prior to your official cancer diagnosis? | | | | | | | |
| 1-2 | cancer survivor/ patient | *My GP kept telling me that it was a virus and just need to rest and drink plenty of water. Went to a walk-in clinic and asked for a CT scan to be performed as was told by GP after asking for CT scan that if I wanted to see specialist it could take three months before I got in* | Delayed diagnosis due to GP attributing symptoms to a virus and challenges in accessing specialists promptly. Had to self-initiate diagnostic scan themselves | delayed diagnosis and limited specialist access  self-initiated diagnostic scan | **Delayed diagnostics due to GP assumptions** | Perceived invalidation of medical concerns  Delayed diagnosis and self-initiation | Perceived delayed diagnosis  GPs dismissive of concerns |
| less than 1 year | family member/ caregiver | *I was told biopsy would be an overnight, maybe two nights, turned into not leaving hospital, so waiting for confirmation diagnosis in at hospital took 2 weeks for full results, was given diagnosis, that then changed several times and got worse each time. think that perhaps waiting although frustrating for patient better than being given false information and having things get worse and worse* | Misleading information about biopsy duration, changing diagnosis, and prolonged wait for confirmation, suggesting the importance of accurate and timely communication. | biopsy misinformation and prolonged confirmation | misleading information and changing diagnosis | Communication and information challenges | (challenges with communication **between the patient and medical team)** |
| 1-2 | cancer survivor/ patient | *GP's clinging to wanting a high PSA prior to any Urologist referral or thought of an MRI as a general indicator as there really is no other definitive method. DRE's are not popular any longer BUT it was that 50% success rate as only 50% felt that detected mine. lack of continuity of PSA component of general blood tests. If pathology group changed, continuity with previous readings (e.g. SA Pe[a]th have last 3 or 4) & I am sure if change Medical Clinic, that complete history not transferred. I find I am the only reliable keeper of that history when it comes to identifying slow but progressive increase (mine now 4.8 BUT I can say in 2010 it was 1.1 & I can see the marked increase from about 2016. My Health / my Gov great idea but not supported to be the success it could have been* | Challenges related to PSA testing criteria and lack of continuity in medical records, emphasising the need for better indicators and seamless record transfer. | challenges with PSA testing and medical record continuity | PSA testing criteria challenges (conflicting views).   Challenges with PSA testing criteria | Communication and information challenges | Challenges in communication **between members of the medical team** |
| 5-10 | family member/ caregiver | *Waiting more than 6 weeks to see GP about a health complaint. No services available in regional areas. Turned out to be cancer.* | Difficulty accessing healthcare services, especially in regional areas, leading to delayed medical attention. | limited access to healthcare services in regional areas. delayed diagnosis | difficult accessing healthcare in regional areas  delayed diagnosis | Practical and Logistical barriers  Perceived delayed diagnosis | Long GP wait times  Poor access to healthcare services in regional areas |
| more than 10 years | family member/ caregiver | *She saw multiple specialists for pain the shoulder, but all diagnosed a sporting injury without examining her. Eventually the growth burst through the muscle, continually growing. Nothing was done for her as the doctors believed the primary site was elsewhere but never located.* | Multiple specialists failing to accurately diagnose and locate the primary site of the condition | No examination and late diagnosis. | No examination and late diagnosis. | Perceived invalidation of medical concerns | Delayed diagnosis |
| 1-2 | family member/ caregiver | *Timely access to a GP. seeing a different medical officer each appointment (different person from initial consult to surgery to follow up)* | Challenges in timely access to GP and inconsistent healthcare personnel during the consultation process. | No timely access and lack of continuity in medical care | limited coordination among healthcare providers | Limited Care Coordination  Practical and Logistical Barriers | Healthcare coordination issues Long GP wait times |
| more than 10 years | family member/ caregiver | *Health practitioners being unaware of services. Information not being communicated between his multiple practitioners (he had multiple conditions, cancer and other). Information, including discharge summaries being late and incorrect. Support services not being available out of hours, on weekends and over Christmas break. In hospice and hospitals, some nursing shifts being amazing with care (especially bowel care), other shifts not giving it the attention it needed to keep him comfortable and safe.* | Concerns about coordination among healthcare providers, delayed and incorrect information sharing, and limited availability of support services, indicating the need for better healthcare coordination and extended support hours. | limited healthcare coordination and support services availability | health care coordination issues | Communication and information challenges  Poor Care Coordination | Challenges in communication **between members of the medical team**  coordination among health care providers  miscommunication within/ among healthcare team. |
| 1-2 | cancer survivor/ patient | *From the ripple effects of diagnosis and treatment. Information about prostate cancer and about the effects of hormone treatment. Information about pathways of treatment* | A gap in information about prostate cancer and its treatment pathways, suggesting a need for better patient education. | lack of information on prostate cancer and treatment pathways | limited information about cancer provided | Perceived inadequate information provision | (challenges with information provision **between the patient and medical team)** |
| less than 1 year | cancer survivor/ patient | *Doctor didn't follow up properly and was quite dismissive of symptoms* | Inadequate follow-up and dismissive attitude of a doctor regarding symptoms. | insufficient follow-up and dismissive medical care | insufficient follow-up and dismissive medical care | Perceived invalidation of medical concerns | GP dismissive of patient concerns |
| less than 1 year | cancer survivor/ patient | *I think several GPs missed the opportunity to give me an early diagnosis due to my age (relatively young) and overall good health. My severe lack of iron was not explored, just an assumption that it was due to heavy periods/hormones.* | Potential missed early diagnosis due to assumptions based on age and overall health. | missed diagnosis due to assumptions | missed diagnosis due to assumptions | Perceived invalidation of medical concerns | Perceived delayed diagnosis |
| 2-5 | cancer survivor/ patient | *It took 11 months of excruciating back pain, including six trips to emergency and multiple trips to GPs and then me losing the use of my legs before doctors finally listened to me and investigated what was wrong. I begged for an MRI and was told it was too expensive, one GP finally caved but was so annoyed she wrote lower lumbar (it was more middle to upper back) with no other info, so the imaging team didn’t know what they were meant to be looking at and they missed my tumour by 1 cm. When I lost the use of my legs they did another MRI of my whole back and found a huge tumour on my T10 vertebrae.* | Lengthy delay in diagnosis due to medical negligence of concerns and inadequate imaging. | delayed diagnosis due to perceived medical invalidation of concerns | Perceived invalidation of medical concerns  Delayed diagnosis | Perceived invalidation of medical concerns  Delayed diagnosis | GPs dismissive of medical concerns  Perceived delayed diagnosis |
| 5-10 | family member/ caregiver | *The GP in a rural medical practise had poor communication skills. The client was suffering from dementia and also had poor communication skills. The GP was probably of a culture that touching a woman patient was not done so a thorough medical examination was never performed. Even when the GP had a consult with 2 family members regarding the elderly patient's declining condition the GP denied the concerns and didn't suggest to do any investigations or prescribe medications to relieve pain. An ACAT assessor suggested getting a second opinion with a different Doctor. After the second opinion and the beginning of tests that were appropriate it became obvious that a cancer diagnosis came too late for survival of the patient. The patient was disadvantaged for being an elderly woman in a rural town with a culturally inadequate male GP.* | Challenges related to communication and cultural aspects in a rural healthcare setting, affecting timely diagnosis and appropriate care. | communication and cultural challenges in rural healthcare |  | Perceived invalidation of medical concerns  Delayed diagnosis | GP dismissive of patient concerns  Poor communication (between patient and the medical team) |
| 2-5 | cancer survivor/ patient | *I experienced a growing level of fatigue and severe night sweats before my diagnosis. I went to several GPs but doctors put it down to my earlier diagnosis of prostate cancer. I had to beg for a scan.* | Misattribution of symptoms to a prior diagnosis, delaying the identification of a new cancer | misattribution of symptoms  delayed diagnosis | misattribution of symptoms  delayed diagnosis | Perceived invalidation of medical concerns | Had to self-initiate diagnostic imaging.  Perceived delayed diagnosis  GPs dismissive of patient concerns  Misattribution of symptoms |
| 5-10 | cancer survivor/ patient | *The initial challenge was trying to get the medical fraternity interested that there was an underlying health issue. It took 9 months and numerous doctors to get someone to show some interest and test.* | Initial challenge in convincing medical professionals of underlying health issues and prolonged diagnosis timeline. | struggles in gaining medical attention and diagnosis | perceived invalidation of medical concerns | Perceived invalidation of medical concerns | misattribution of symptoms  Perceived delayed diagnosis |
| 5-10 | cancer survivor/ patient | *GP not picking up abnormalities in routine blood results and requesting further testing. GPs understanding that the cancer I have is not only 'an old persons cancer'* | Challenges related to GP understanding and misconceptions about the type of cancer, implying a need for more accurate awareness. | GP understanding and misconceptions about the condition | GP understanding and misconceptions about the condition | Perceived invalidation of medical concerns & Delayed Diagnosis | Assumptions by medical specialists  Misattribution of symptoms |
| 5-10 | cancer survivor/ patient | *Original misdiagnosis* | Initial assessment resulting in a misdiagnosis | misdiagnosis in initial assessment | misdiagnosis in initial assessment | Misattribution of symptoms | Misattribution of symptoms |
| 5-10 | cancer survivor/ patient | *It was assumed my medical specialists that I had osteoporosis after collapsing with lumbar fractures* | Assumptions by medical specialists leading to a misdiagnosis. | assumptions leading to misdiagnosis | assumptions leading to misdiagnosis | Misattribution of symptoms | Misattribution of symptoms  Assumptions by medical specialists |
| 5-10 | cancer survivor/ patient | *I was originally misdiagnosed and ignored* | Experience of misdiagnosis and feeling ignored in the healthcare process. | Misdiagnosed and ignored | Misdiagnosed and ignored | Perceived invalidation of medical concerns | GPs dismissive of medical concerns |

**Supplementary 3**: Example of Quotations supporting Analysis

N.B. This list is not exhaustive and includes examples.

| Theme/Component | Examples of Quotations/ Evidence |
| --- | --- |
| The level of an individual’s care needs (and thus their need for cancer navigation support) is dynamic and can evolve during one’s patient's journey. | - The same individuals requesting different levels of support at different time points - Different individuals requesting different levels of support at the same time point |
| All individuals affected by cancer share a baseline level of informational and resource needs and barriers to care, necessitating a minimum level of informational navigation support (information to support navigation) | **Lack of awareness of available services**   - *“I know there are many support services available, it is somewhat challenging to identify them and organise them” –* **survey respondent***.”* - *“it was seamless for us because we were aware of services and all the staff gave us plenty of information” –* **survey respondent** - *“Health professionals are unaware of services. Information is not being communicated between different practitioners. Information during discharge summaries are late and incorrect” -***survey respondent** - *“Yeah, yeah. And I think ‘awareness’ is for me, it's critical because it's awareness of services that are available, so that people can access them. Awareness of things like (you know because as I said working with the Cancer Council and you know) early detection, all that sort of thing. And I think also as I said, you know people being aware that there is support out there, knowing this is where you go to or you know, I think that's really important.” –* **Interviewee 1** - *”at the beginning [at diagnosis] especially, that initial period of quickly developing detailed knowledge of treatments & services to adequately discuss, assess & choose treatments & utilise available services was challenging. Had to educate myself, hard to find this information”.* ***-* Survey respondent** - *“If we had a person helping you navigate the system, they should be able to connect you to organisations that are related to yourself. Cancer Council for example has good holistic support.” –* **Interviewee 12**   **Felt more empowered (and reported no gaps in care) when provided with information**   - *“My husband and I had enough information about where to access everything. His doctor was really good… we really didn’t need to engage anyone extra" -***survey respondent** - *“it was seamless for us because we were aware of services and all the staff gave us plenty of information” –* **survey respondent** - *I was very lucky with good advice from Dr [redacted] from the clinical trial, because he would meet with me. –* **survey respondent**   **Lack of information on symptom management, care management**   - *“information and education on how to apply for NDIS”* - *“There needs to be more information given to us from the start, so we know what’s happening rather than me having to research everything by myself.” –* **Interviewee 11** - *…but I thought that they [oncologists] would maybe have given me more of a rounded look about living with this cancer. –* - *“Just information about the dangers of not having scans or biopsies.”* - *if I didn’t do my research I wouldn’t know about these trials. There needs to be more support in finding suitable trials for different cancer types.” –* **Interviewee 12** - *“we need more accessible public information about symptoms”* - *“I want access to test results, its so hard to manage everything without”* **– survey respondent** - *“our families need access to our care plans’* – **survey respondent** - *I was very lucky with good advice from Dr [redacted] from the clinical trial, because he would meet with me. But I found there was limited communication between individual doctors which was frustrating for me. I ended up getting removed from the trial as I was no longer eligible. I was reading the handbooks from Cancer Council SA and my wife always comes to my consults and asks questions and checks up on things. –* **survey respondent** - *I also feel the GPs need to be up to date with the clinical trials. That would have been helpful –* **Interviewee 15** - *It already took forever to get my biopsy done and then during my radiation treatment, I was given nothing, no information. What are the side effects, will I get crook after my treatment, nothing. I can imagine with side effects as well, everyone is different. I get that but there needs to be someone’s job to tell me this stuff. –* **Interviewee 18** - *And the fact that my PSA levels aren’t transferred to other doctors, for example if I changed my pathology group, my complete history was obviously not being transferred. SA pathology only has my last 3 or 4 tests but what about the others? I find I am the only reliable keeper of that history when it comes to identifying slow but progressive increases in my PSA levels. I think my levels are now 4.8. –* **Interviewee 19** |
| Some individuals seek and/or require additional support beyond information and express a desire for peer interaction and community-based navigation support. | **Positive experiences, individuals value peer-based support.**   - *“Yeah, initially we knew nothing about support services or resources but found it out ourselves over a period of time through starting a support group and becoming involved with the PCFA. Doctors should be more willing to advise patients about support groups where information is freely shared and people can share experiences and feel that they are not alone on their cancer journey.”* - *I've got a lot of written information, pamphlets, brochures, all sorts of stuff and that's the issue I had was I got all the written stuff, but I didn't know who to contact because I had questions. You know it took a bit of running around and you know, I spoke to leukaemia foundation. I spoke to cancer council. But I've noticed the double generic, but I need specifics and eventually it was the Myeloma Australia that pointed me in the right direction.* **Interviewee 6** - *The support nurses that we have here in Adelaide are absolutely awesome. And the support groups that we've got that's been just terrific.* **Interviewee 6** - *But having also been member of the Mitchum prostate support group. I realised how great they are. Telling the people who aren't in my situation, and they've either left it too long or I've had publications, and you know it could have turned out a whole lot worse and I guess my ignorance is bliss.* **Interviewee 7** - *“It was nice to be able to talk to people with similar circumstances. And I really valued the support groups run by Myeloma Australia.*” **Survey respondent** - *“The MRF brain support group services have been very helpful. Would be lost without them. However, in regards to advocating with my employer and NDIS they can only provide contact information and don’t contact or advocate on my behalf”* - And I haven't been one person, a person, to take part in these online, you know, support groups. But the nurse from the Leukaemia Foundation did contact me about 1:00. - **Interviewee 10** - *In the early days, I attended the Mitcham prostate group and was provided with booklets that were out of date. –* **Interviewee 15** - *Yeah. I guess my breast cancer nurse was helpful almost like a navigator like you mentioned earlier. I could ask her questions if I was worried about side effects and where to find resources and support groups and things like that. –* ***Interviewee 16*** - *This whole process was too fast and I never really had the chance to become a student of my disease, it was more like get em in and done and move them on as quick as possible. Finding PCFA [Prostate Cancer Foundation Australia] and being in the position with a bit of time on my side allowed be to one step ahead of the game so to speak.*   **Not everybody expressed a desire or need for additional peer-based support**   - *“We have a very supportive community here. We're a very close-knit community. And you know, I just had people that supported us. They bought us meals. They came and sat with [redacted] while I could go out for a little bit and things like that. I'm probably not the best example for needing, you know, using other support services and requiring them.” – Interviewee 1* - *“Yes, I was provided with a support nurse throughout my care but didn’t use her services. I didn’t feel I needed her. But it was good to know that support was there, if I wanted”* - *“Was given the contact details [of* myeloma specialist nurse] *but I never needed to call them.” – Participant 11* - *I didn’t engage with any peer groups, I didn’t feel the need* - *“I don't find the [peer-based support] groups very supportive. They're just a group that gather because they're a group. OK, because maybe what?”).* - *“My husband and I had enough information about where to access everything. His doctor was really good… we really didn’t need to engage anyone extra”* |
| Certain populations have disparate cancer outcomes, diverse experiences of cancer care, and complex care needs, signalling a heightened requirement for more intensive navigation support | Amalgamation of the perspectives shared by consumers throughout the consultation process   - "*I didn’t receive any support [for neuropathy]. I had no idea what I was doing. –* **Survey respondent** - *It needs to be recognised that a cancer diagnosis affects the whole family. The transplant coordinators at the (Royal Adelaide Hospital) RAH weren’t too accommodating as I wasn’t too keen on hospital at home as I didn’t want my kids to see but I wasn’t given a choice. I had to do it at home-* **Interviewee 14** - *I would have liked it if there was a system we could call. Remember, during COVID, if you ever rang the hospital to ask if you should come in, they put you through a system, so if you had a question that a nurse could answer, they would put you through. But if it, yeah. But if it wasn't relevant, they wouldn't put you through. So I think that would be helpful. It needs to be efficient*- |
